# Analysis of the Role of KLF4 in the Regulation of Porcine Epidemic Diarrhea Virus Infection

**DOI:** 10.3390/ani15162343

**Published:** 2025-08-11

**Authors:** Haifei Wang, Yajing Zhou, Shanshen Gu, Mengke Feng, Jie Wang, Jian Jin, Xiaoguo Huang, Shenglong Wu, Wenbin Bao

**Affiliations:** 1 Key Laboratory for Animal Genetics, Breeding, Reproduction and Molecular Design, College of Animal Science and Technology, Yangzhou University, Yangzhou 225009, China; hyfiwang@yzu.edu.cn (H.W.); yajingzhoucindy@163.com (Y.Z.); gshanshen@163.com (S.G.); mengke202504@163.com (M.F.); wangjie4228@163.com (J.W.); jianj1127@163.com (J.J.); slwu@yzu.edu.cn (S.W.); 2 Jiangsu Fenghua Agricultural Group Co., Ltd., Changzhou 213148, China; klhxg@163.com; 3 Joint International Research Laboratory of Agriculture & Agri-Product Safety, Yangzhou University, Yangzhou 225009, China

**Keywords:** PEDV, KLF4, gene editing, transcriptome, virus resistance

## Abstract

Porcine epidemic diarrhea virus (PEDV) is a primary pathogen responsible for viral diarrhea in swine. The identification of host resistance genes and key regulatory elements represents a critical prerequisite for developing novel control strategies. Krüppel-like factor 4 (KLF4), a multi-functional transcription factor, is known to regulate pathogenic infections; however, its specific roles in PEDV infection remain largely undefined. In this study, we found that KLF4 expression was upregulated following PEDV infection. Next, we constructed KLF4 knockout and overexpression cells and infected them with PEDV. The results show that viral RNA and protein expression levels and infectious viral titers were significantly enhanced in PEDV-infected KLF4 knockout cells compared to infected wild-type cells. In contrast, PEDV infection levels were significantly decreased in KLF4 overexpression cells relative to control cells. Transcriptomic analysis reveals that KLF4 significantly influences the expression of genes involved in key signaling pathways, including PI3K/Akt and MAPK. Overall, our findings elucidate the functional roles and underlying mechanisms of KLF4 during PEDV infection, offering valuable molecular targets for PEDV prevention and control.

## Simple Summary

Porcine epidemic diarrhea virus (PEDV) causes severe diarrhea in pigs, and identifying host resistance genes is crucial for developing control strategies. This study investigated the role of Krüppel-like factor 4 (KLF4), a transcription factor, during PEDV infection. The results show that KLF4 expression increased after PEDV infection. Knockout of KLF4 enhanced viral RNA/protein expression levels and infectious titers, while KLF4 overexpression reduced PEDV infection. Transcriptomic analysis reveals that KLF4 regulates key pathways like PI3K/Akt and MAPK, influencing host responses to PEDV. Together, these findings highlight KLF4 as a critical host factor inhibiting PEDV, highlighting it as a promising target for antiviral strategies.

## 1. Introduction

Porcine epidemic diarrhea (PED), one of the most devastating swine enteric diseases, is caused by the porcine epidemic diarrhea virus (PEDV) and manifests an acute clinical manifestation. PED is particularly lethal to newborn piglets and causes mortality rates reaching 100% due to severe fluid loss from characteristic vomiting and watery diarrhea [1,2]. First reported in the UK in 1971, PED subsequently spread across Europe and Asia, establishing endemic circulation in pig herds [3]. In 2010, a highly pathogenic PEDV strain emerged in China and rapidly disseminated across Asian countries. Epidemiological investigations confirmed fecal–oral transmission as the primary route among pigs. Moreover, contaminated animal feed and dietary supplements have been identified as significant fomites in disease dissemination [4,5]. Studies further revealed that the virus infects the nasal epithelium to enter the bloodstream, ultimately causing intestinal infection [6]. Therefore, it is crucial to develop systematic prevention and control strategies for PEDV infection.

Krüppel-like factor 4 (KLF4) is a pleiotropic transcription factor involved in regulating multiple cellular processes, including proliferation, apoptosis, and tissue morphogenesis [7,8]. KLF4 can activate or inhibit gene transcription through post-translational modifications such as acetylation and methylation [9]. Additionally, KLF4 induces reactivation of the Epstein–Barr virus by binding to and activating the BZLF1 gene promoter [10]. KLF4 also inhibits IRF3 recruitment, thereby inhibiting IFN-β production and impairing the antiviral immune response following vesicular stomatitis virus stimulation [11]. Significant upregulation of KLF4 expression occurs in the intestines of PEDV-infected pigs and PEDV-treated intestinal porcine epithelial cells [12,13]. These findings suggest that KLF4 participates in host immune responses triggered by pathogenic stimuli; however, its specific function during PEDV infection remains to be elucidated.

In this study, to investigate the role of KLF4 in PEDV infection, we utilized porcine intestinal epithelial cells (IPEC-J2) as a study model. KLF4 knockout and overexpressing cells were established and infected with PEDV. Meanwhile, RNA-seq was performed to identify genes associated with KLF4 regulation during PEDV infection. These results expand our understanding of the role of KLF4 in the cellular response to PEDV infection and provide new molecular targets for developing antiviral strategies against PEDV.

## 2. Materials and Methods

### 2.1. Cells, Viruses, and Reagents

Porcine intestinal epithelial cells (IPEC-J2) and African green monkey kidney cells (Vero) were maintained in our laboratory. Cells were grown in Dulbecco’s Modified Eagle’s Medium (DMEM; Gibco, Grand Island, NE, USA) supplemented with 10% fetal bovine serum (FBS; Gibco, Grand Island, NE, USA) and 1% penicillin/streptomycin (Solarbio, Beijing, China) at 37 °C with 5% CO_2_. The PEDV strain CV777 was stored in our laboratory. Antibodies used in this study included anti-PEDV N (DA0124, Youlong biotech, Shanghai, China), anti-KLF4 (sc-166238, Santa Cruz, CA, USA), anti-GAPDH (25074-1-AP, Proteintech, Wuhan, China), anti-HSP90 (60318-1-Ig, Proteintech, Wuhan, China), goat anti-rabbit IgG (CW0103S, CWBIO, Taizhou, China), and goat anti-mouse IgG (CW0102S, CWBIO, Taizhou, China).

### 2.2. Generation of Gene Knockout and Overexpression Cells

For KLF4 knockout in IPEC-J2 cells, sgRNAs targeting the porcine KLF4 gene were designed using CHOPCHOP [14] and cloned into the pGK1.2 vector (Table S1). The recombinant knockout vectors were transfected into IPEC-J2 cells using Lipofectamine 3000 (Invitrogen, Waltham, MA, USA) following the manufacturer’s protocol. After 48 h post-transfection, positive clones were selected with 2 μg/mL puromycin for 7 days, and monoclonal cells were subsequently obtained by limited dilution. KLF4 knockout was confirmed by Sanger sequencing of PCR-amplified genomic regions and by Western blot analysis of KLF4 protein expression.

For KLF4 overexpression in IPEC-J2 cells, the full-length coding sequence of KLF4 was synthesized and cloned into the pcDNA3.1-3xFlag vector using the ClonExpress II One Step Cloning Kit (Vazyme, Nanjing, China). The overexpression vectors were transfected into IPEC-J2 using the same method as for the knockout vectors. After 48 h of post-transfection, geneticin (G418, 4 μg/mL) was added to filter positive clones. KLF4 expression was measured by qRT-PCR and Western blot assays.

### 2.3. PEDV Infection

According to the experimental requirements, cells were seeded in appropriate culture plates and maintained overnight at 37 °C with 5% CO_2_. PEDV was inoculated into the test wells with an infection multiplicity (MOI) of 1. After 2 h of viral adsorption, cells were washed three times with PBS and then cultured in DMEM containing 2% FBS and 4 µg/mL trypsin at 37 °C with 5% CO_2_. Cell samples were collected at designated time points post-infection for subsequent analysis.

### 2.4. RNA Extraction and Quantitative Real-Time PCR (qRT-PCR) Assay

Cellular RNA was extracted using Trizol reagent (Takara Biotechnology, Dalian, China) and subsequently reverse-transcribed into cDNA using the HiScript III RT SuperMix for qPCR (Vazyme, Nanjing, China) in accordance with the recommended procedures. Gene expression was quantified by qRT-PCR using the SuperStar Universal SYBR Master Mix reagent (CWBIO, Taizhou, China). Each reaction consisted of 1 μL of cDNA template, 10 μL of SYBR Green Mixture, 1 μL of each primer, 0.4 μL of 50× ROX Reference Dye II, and 6.6 μL of deionized water. The thermal cycling conditions were as follows: 95 °C for 15 s, followed by 40 cycles of 95 °C for 5 s and 60 °C for 30 s. Data were normalized to GAPDH expression. The sequence of primers used in the qRT-PCR assay is shown in Table S2.

### 2.5. SDS-PAGE and Western Blot Analysis

Cells were washed twice with PBS and lysed on ice using RIPA buffer containing a protease inhibitor cocktail. Protein concentrations were determined using the Enhanced BCA Protein Assay Kit (Beyotime, Shanghai, China). Lysates were then denatured in 5×SDS-PAGE loading buffer for 10 min. Protein samples were separated by SDS-PAGE and transferred onto polyvinylidene fluoride (PVDF) membranes (Merck Millipore, Darmstadt, Germany). Membranes were blocked with QuickBlock^TM^ Blocking Buffer (Beyotime, Shanghai, China) for 20 min at room temperature. Primary antibodies were incubated with the membranes at room temperature for 2 h, followed by incubation with species-matched secondary antibodies for 1 h at room temperature. Protein bands were visualized using enhanced chemiluminescence (Thermo Fisher Scientific, Waltham, MA, USA) and quantified using Image J 1.8.0 software.

### 2.6. The 50% Cell Culture Infectious Dose (TCID_50_) Assay

Cells were seeded in 96-well plates at a density of 10^4^ cells/well and cultured overnight under standard conditions. The viral solution was serially diluted in a ratio of 1:9 by adding 10 µL of viral solution to 90 µL of DMEM containing 2% FBS. The diluted viral solutions were added to wells in columns one through six of the plate, using a fresh pipette tip for each column. Plates were incubated at 37 °C with 5% CO_2_ for 7 d, and cytopathic effects (CPE) were monitored daily under an inverted microscope. Viral titers (TCID_50_ per mL) were calculated using the Reed–Muench method.

### 2.7. Indirect Immunofluorescence Assay

Cells were placed in 12-well plates at a density of 10^5^ cells/well and cultured overnight under standard conditions. After 48 h post-PEDV infection, cells were fixed with 4% paraformaldehyde for 30 min at room temperature, followed by permeabilization with 0.5% Triton X-100 for 15 min. Cells were then blocked with 5% BSA at room temperature for 1 h. The PEDV N antibody was incubated with the cells at 4 °C overnight, followed by incubation with species-matched fluorescent secondary antibodies at room temperature for 1 h. Nuclei were stained with DAPI for 5 min, and images were acquired using a wide-field fluorescence microscope (Leica, Wetzlar, Germany).

### 2.8. Transcriptome Data Analysis

Three biological replicates were prepared for each group: wild-type (WT), KLF4-knockout (KO), PEDV-infected WT, and PEDV-infected KLF4-KO. Total RNA was extracted using Trizol reagent (Takara Biotechnology, Dalian, China). Transcriptome sequencing was conducted at Novo Gene Technology Co., Ltd. (Beijing, China) using the Illumina HiSeq-PE150 platform. Low-quality reads, including those with adapter contamination or base quality scores below 20, were removed to obtain clean reads. Gene expression levels were quantified as fragments per kilobase of transcript per million mapped reads (FPKMs), with an FPKM value greater than 1 considered indicative of gene expression. Clean reads were aligned to the Sus scrofa reference genome (Sscrofa11.1) using TopHat2 [15]. Differentially expressed genes (DEGs) were identified using DESeq2 [16], with DEGs defined as those with |log2 fold change| ≥ 1 and an adjusted *p*-value ≤ 0.05.

### 2.9. Functional Annotation of DEGs

Gene ontology (GO) and KEGG pathway enrichment analyses of the DEGs were performed using the clusterProfiler package in R 4.4.0 software [17]. The Benjamini and Hochberg method was used to adjust the *p*-values for multiple testing, and GO terms and pathways with an adjusted *p*-value < 0.05 were considered statistically significant.

### 2.10. Statistical Analysis

All data are presented as mean ± standard deviation (SD). Statistical analyses were performed using GraphPad Prism software (version 8). Student’s *t*-test was used to compare statistical significance between two groups. For comparisons involving more than two groups, one-way analysis of variance (ANOVA) followed by Tukey’s multiple comparisons post-tests was performed. A *p*-value < 0.05 was considered statistically significant. * *p* < 0.05, ** *p* < 0.01, *** *p* < 0.001.

## 3. Results

### 3.1. KLF4 Expression Is Increased in PEDV-Infected Host Cells

**Figure 1:**
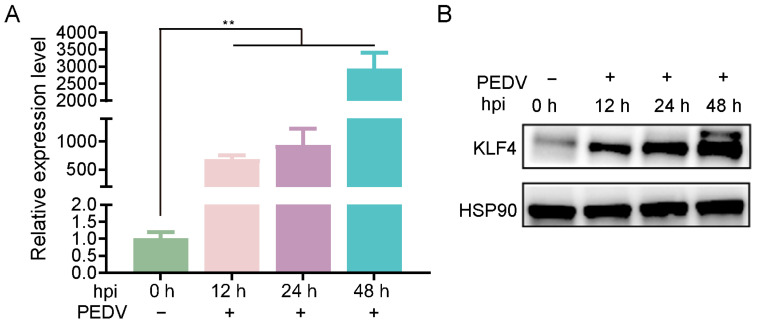
KLF4 expression of mRNA (**A**) and protein (**B**) levels in PEDV-infected cells at various time points (0, 12, 24, and 48 h). hpi: hours post-infection; PEDV: porcine epidemic diarrhea virus. ** *p* < 0.01.

To investigate the potential roles of KLF4 in cellular responses to PEDV infection, we infected IPEC-J2 cells with PEDV and quantified KLF4 expression by qRT-PCR and Western blot assays. The results show that KLF4 mRNA expression was significantly upregulated in PEDV-infected cells and peaked at 48 h post-infection (Figure 1A). Western blot analysis further confirms the increased expression of KLF4 protein in PEDV-infected cells (Figure 1B). These findings collectively demonstrate that PEDV infection obviously induces KLF4 expression in IPEC-J2 cells.

### 3.2. Knockout of KLF4 Promotes PEDV Replication in IPEC-J2 Cells

**Figure 2:**
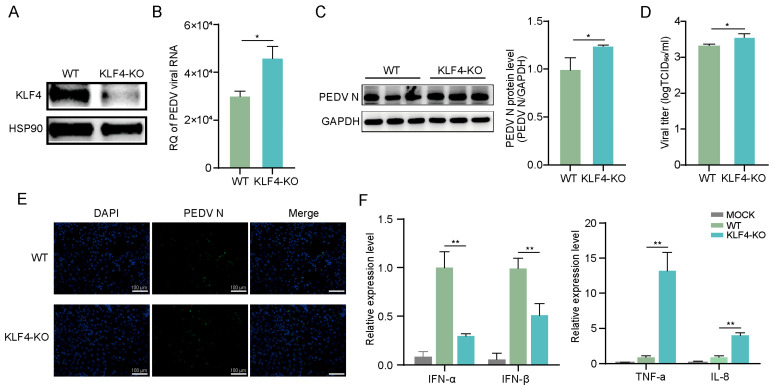
KLF4 knockout promotes PEDV replication and modulates host immune response. (**A**) Western blot analysis of KLF4 in KLF4-KO and WT cells. (**B**) Viral RNA levels in PEDV-infected KLF4-KO and WT cells at 48 hpi. (**C**) Western blot analysis of PEDV N protein in PEDV-infected KLF4-KO and WT cells at 48 hpi. (**D**) Infectious viral titers determined by TCID_50_ in supernatants of PEDV-infected KLF4-KO and WT cells at 48 hpi. (**E**) Immunofluorescence microscopy of PEDV N protein (green) at 48 hpi in PEDV-infected KLF4-KO and WT cells. Nuclei were counterstained with DAPI (blue). Scale bars: 100 µm. (**F**) Relative expression of interferons and pro-inflammatory cytokines in MOCK, PEDV-infected KLF4-KO, and WT cells at 48 hpi. KLF4-KO: KLF4 knockout cells; WT: wild-type cells; MOCK: mock-infected cells. Data are presented as mean ± SD (*n* = 3). * *p* < 0.05, ** *p* < 0.01.

To investigate the function of KLF4 during PEDV infection, we established KLF4 knockout IPEC-J2 cells using CRISPR/Cas9 (Figure S1). Western blot analysis shows that KLF4 protein was nearly undetectable in the knockout cells (Figure 2A). Next, we infected KLF4 knockout and wild-type cells with PEDV and assessed viral infection. Viral RNA and protein expression levels were significantly increased in KLF4 knockout cells compared to those in wild-type cells (Figure 2B,C). Moreover, infectious viral titers in the supernatants of KLF4 knockout cells were significantly higher than those in wild-type cells (Figure 2D). Immunofluorescence analysis further demonstrates enhanced viral infection in KLF4 knockout cells relative to wild-type cells (Figure 2E). Additionally, qRT-PCR shows that the expression levels of interferons (IFN-α and IFN-β) were noticeably downregulated in KLF4 knockout cells, while those of pro-inflammatory cytokines (IL-8 and TNF-α) were significantly increased (Figure 2F). These results indicate that KLF4 depletion promoted PEDV infection in IPEC-J2 cells and led to increased interferon response and an increased pro-inflammatory cytokine response.

### 3.3. Overexpression of KLF4 Suppresses PEDV Replication in IPEC-J2 Cells

**Figure 3:**
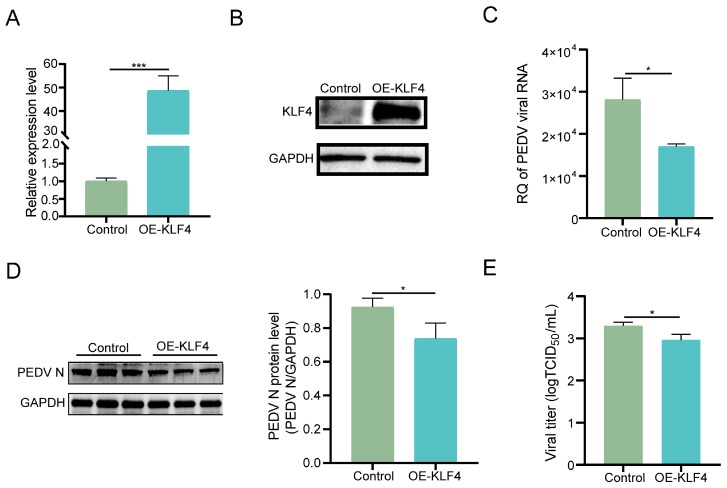
KLF4 overexpression inhibits PEDV infection in host cells. KLF4 mRNA (**A**) and protein (**B**) expression levels in overexpression and control cells. Viral RNA (**C**) and protein (**D**) expression levels in PEDV-infected KLF4 overexpression and control cells at 48 hpi. (**E**) Infectious viral titers determined by TCID_50_ in supernatants of PEDV-infected KLF4 overexpression and control cells at 48 hpi. OE-KLF4: KLF4 overexpression cells; Control: cells transfected with empty control vectors. Data are presented as mean ± SD (*n* = 3). * *p* < 0.05, *** *p* < 0.001.

To verify the function of KLF4 during PEDV infection, we further generated KLF4 overexpression cells. qRT-PCR and Western blot analyses confirm dramatically higher expression of KLF4 at both mRNA and protein levels in the overexpression cells (Figure 3A,B). Subsequently, we infected them with PEDV and observed that viral RNA and protein expression were significantly decreased in KLF4 overexpression cells compared to controls (Figure 3C,D). In addition, infectious viral titers in the supernatants of KLF4 overexpression cells were significantly lower than those of controls (Figure 3E). Overall, these results support the critical role of KLF4 in suppressing PEDV infection in IPEC-J2 cells.

### 3.4. Transcriptomics Analysis of KLF4 Knockout Cells Infected with PEDV

**Figure 4:**
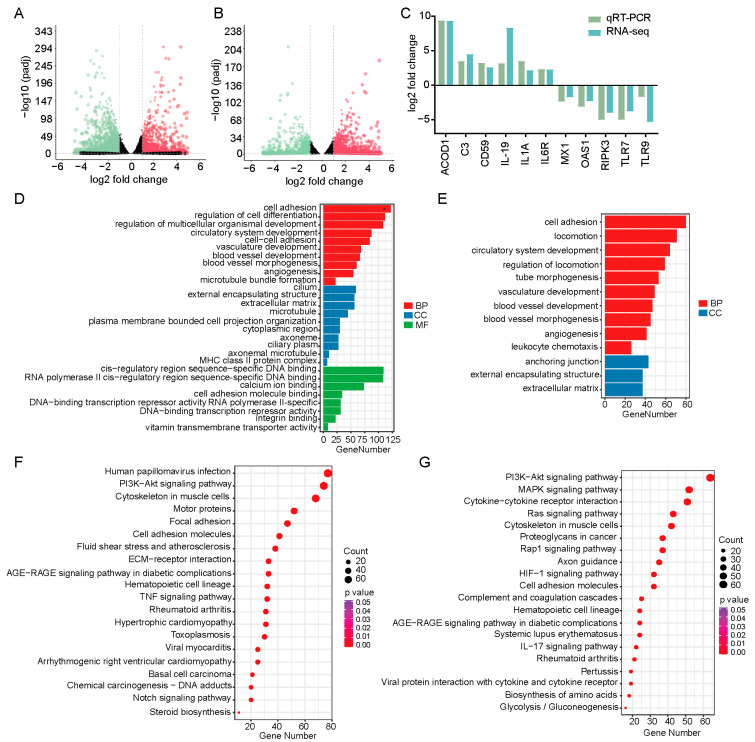
Transcriptomics analysis in KLF4 knockout and wild-type cells with or without PEDV infection. (**A**) Volcano plots of DEGs between PEDV-infected and mock-infected cells. (**B**) Volcano plots of DEGs between PEDV-infected KLF4 knockout and wild-type cells. The red and green dots represent significantly upregulated and downregulated genes, respectively. (**C**) Expression changes in DEGs quantified by RNA-seq and qRT-PCR. (**D**,**E**) Gene ontology analysis for DEGs shown in (**A**,**B**), respectively. The red, blue, and green bars indicate biological process, cellular component, and molecular function, respectively. (**F**,**G**) KEGG pathways analysis for DEGs shown in (**A**,**B**), respectively.

To characterize the potential biological processes mediated by KLF4 during PEDV infection, we performed transcriptomic analysis on KLF4 knockout and wild-type cells with or without PEDV infection. After quality control, an average of 43.4 million clean reads per sample were obtained, with over 91.79% uniquely mapped to the pig genome (Table S3). Differential expression analysis identifies 3588 DEGs between PEDV-infected and mock-infected cells, of which 2124 were downregulated and 1464 were upregulated (Figure 4A, Table S4). Moreover, 2295 DEGs were identified between PEDV-infected KLF4 knockout and wild-type cells, of which 1372 were upregulated and 923 were downregulated (Figure 4B, Table S5). To validate DEG expression changes, we randomly selected 11 DEGs for qRT-PCR quantification. The results show consistent expression trends with the RNA-seq data, indicating the high reliability and accuracy of our transcriptomic analysis (Figure 4C).

To investigate the functions of the DEGs, we performed gene ontology and KEGG pathway enrichment analyses. GO analysis shows that DEGs between PEDV-infected and mock-infected cells were significantly enriched in categories, including cell adhesion, regulation of cell differentiation, and regulation of multicellular organismal development (Figure 4D; Table S6). For DEGs between PEDV-infected KLF4 knockout and wild-type cells, significant GO enrichment was observed in cell adhesion, locomotion, and circulatory system development (Figure 4E; Table S7). KEGG pathway analysis reveals that DEGs between PEDV-infected and mock-infected cells were significantly enriched in pathways such as human papillomavirus infection, PI3K-Akt signaling pathway, and cytoskeleton in muscle cells (Figure 4F; Table S8). Furthermore, DEGs between PEDV-infected KLF4 knockout and wild-type cells show significant enrichment in pathways, including the PI3K-Akt signaling pathway, the MAPK signaling pathway, and cytokine–cytokine receptor interaction (Figure 4G; Table S9).

## 4. Discussion

KLF4 is a well-established transcription factor regulating antiviral immune response. In this study, we observed increased PEDV infection in KLF4 knockout cells but decreased infection in KLF4 overexpression cells, indicating an important role for KLF4 in the cellular response to PEDV infection. In addition, KLF4 knockout led to decreased antiviral interferon responses, alongside increased pro-inflammatory cytokine responses. These findings indicate a weaker interferon response with KLF4 depletion, which may facilitate PEDV replication in host cells. Previous in vivo studies have identified roles for KLF4 in modulating intestinal epithelial cell morphology and function [18]. Given that PEDV primarily infects small intestinal epithelial cells, this highlights the potential diverse functions of KLF4 in regulating PEDV infection. Here, we identified an inhibitory effect of KLF4 on PEDV replication in porcine intestinal epithelial cells. Future in vivo studies investigating the role of KFL4 in regulating PEDV infection will provide further functional evidence. Inhibiting viral protein synthesis and promoting viral degradation are key host antiviral strategies [19]. While we reveal the inhibitory effect of KLF4 on PEDV replication, whether KLF4 directly binds to PEDV proteins to suppress virus replication warrants further investigation.

Inhibition of the PI3K-Akt signaling pathway was recently shown to suppress SARS-CoV-2 replication [20]. PEDV nonstructural protein 6 can induce autophagy via the PI3K-Akt pathway to promote virus replication [21]. Additionally, the PI3K-Akt pathway has been regarded as a molecular target for inhibiting PEDV infection [22]. Our transcriptomic analysis reveals significant enrichment of DEGs in the PI3K-Akt pathway. Importantly, a majority of these DEGs showed opposite expression changes when comparing PEDV-infected wild-type cells to PEDV-infected KLF4 knockout cells, underscoring the regulatory role of KLF4 during infection. IL-6 and IL-6R, as upstream activators of the PI3K/AKT pathway [23], were significantly upregulated in PEDV-infected KLF4 knockout cells. Moreover, upregulated expression of PIK3R1 [24] that encodes critical components of the PI3K/AKT pathway was detected in these cells. Concurrently, upregulated expression of the downstream effector FOXO3 [25] was also observed. Collectively, these findings indicate that KLF4 modulates PEDV infection, potentially through regulating the expression of key genes within the PI3K/AKT pathway.

The MAPK signaling pathway is a critical cellular cascade activated by multiple viruses to regulate viral replication [26]. SARS-CoV-2 hijacks the p38/MAPK pathway to promote its replication [27]. African swine fever virus infection activates MAPK signaling to facilitate replication [28], and MAPK pathway activation contributes to PEDV replication [29]. Our transcriptomic analysis reveals significant enrichment of the MAPK pathway by DEGs between PEDV-infected KLF4 knockout and wild-type cells, with a majority of these DEGs showing upregulation. Several genes, including MAP3K1, MAPK13, JUN, and DUSP1, that encode key proteins of the MAPK pathway [30] exhibited significantly increased expression in PEDV-infected KLF4 knockout cells. These findings indicate a potential mechanism whereby KLF4 regulates PEDV replication by affecting MAPK pathway activity. As KLF4 is a multifunctional transcription factor, whether it directly binds promoters of these genes to regulate their expression needs further investigation.

## 5. Conclusions

In summary, our study identifies KLF4 as a critical host factor inhibiting PEDV infection. Transcriptomic analysis further indicates that KLF4 suppresses PEDV infection by regulating genes involved in the PI3K-Akt and MAPK signaling pathways. These findings elucidate the novel functions and underlying mechanisms of KLF4 in modulating PEDV infection, underscoring its potential as a molecular target against PEDV.

## Data Availability

The datasets of this study are available from the corresponding author, Wenbin Bao (wbbao@yzu.edu.cn), upon reasonable request.

## Supplementary Materials

The following supporting information can be downloaded at: https://www.mdpi.com/article/10.3390/ani15162343/s1, Figure S1: Establishment of KLF4 knockout cells; Table S1: Sequences of the sgRNAs targeting the KLF4 gene; Table S2: Primer sequences for qRT-PCR amplification of target genes; Table S3: Statistics for RNA-seq data; Table S4: DEGs between PEDV-infected and mock-infected cells; Table S5: DEGs between PEDV-infected KLF4 knockout and wild-type cells; Table S6: Functional categories significantly enriched by DEGs between PEDV-infected and mock-infected cells; Table S7: Functional categories significantly enriched by DEGs between PEDV-infected KLF4 knockout and wild-type cells; Table S8: Pathways significantly enriched by DEGs between PEDV-infected and mock-infected cells; Table S9: Pathways significantly enriched by DEGs between PEDV-infected KLF4 knockout and wild-type cells.
