# Supplementary material for: Analysis of the Role of KLF4 in the Regulation of Porcine Epidemic Diarrhea Virus Infection
Source: Animals (Basel). 2025 Aug 11;15(16):2343. doi: 10.3390/ani15162343 (PMC12382836; doi:10.3390/ani15162343)
Supplement: Supplementary file 1 [file animals-15-02343-s001.zip › Supplement figures.pdf]

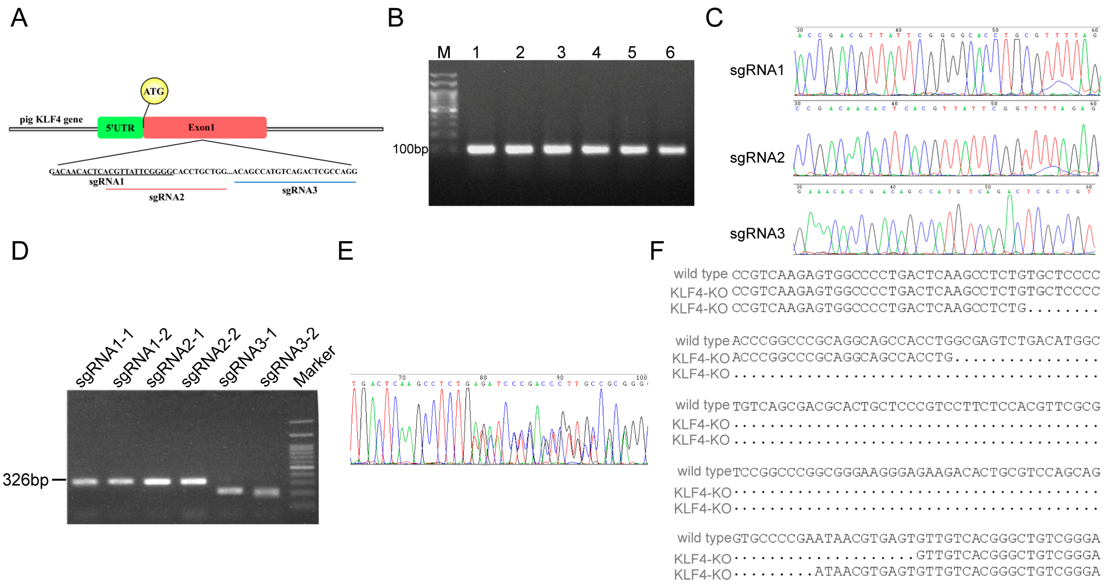

Figure S1. Establishment of KLF4 knockout cells. (A) Scheme of the location of sgRNAs (sgRNA1, sgRNA2, sgRNA3) targeting KLF4. (B) Gel electrophoresis analysis of colony PCR. M: DNA marker, lane1, 2, 3, 4, 5, 6: positive colony bands. (C) Sequencing peak map of colony PCR. (D) Cruiser ™ Enzyme digestion of PCR products of positive knockout cells. (E) Sequencing peak map of mixed positive cell clones. (F) Deleted sequence of the KLF4 knockout cells.
